# BBKNN: fast batch alignment of single cell transcriptomes

**DOI:** 10.1093/bioinformatics/btz625

**Published:** 2019-08-10

**Authors:** Krzysztof Polański, Matthew D Young, Zhichao Miao, Kerstin B Meyer, Sarah A Teichmann, Jong-Eun Park

**Affiliations:** Cellular Genetics, Wellcome Sanger Institute, Hinxton, Cambridge CB10 1SA, UK; Cellular Genetics, Wellcome Sanger Institute, Hinxton, Cambridge CB10 1SA, UK; Cellular Genetics, Wellcome Sanger Institute, Hinxton, Cambridge CB10 1SA, UK; European Molecular Biology Laboratory, European Bioinformatics Institute, Hinxton, Cambridge CB10 1SD, UK; Cellular Genetics, Wellcome Sanger Institute, Hinxton, Cambridge CB10 1SA, UK; Cellular Genetics, Wellcome Sanger Institute, Hinxton, Cambridge CB10 1SA, UK; Theory of Condensed Matter Group, Cavendish Laboratory/Department of Physics, University of Cambridge, Cambridge CB3 0HE, UK; Cellular Genetics, Wellcome Sanger Institute, Hinxton, Cambridge CB10 1SA, UK

## Abstract

**Motivation:**

Increasing numbers of large scale single cell RNA-Seq projects are leading to a data explosion, which can only be fully exploited through data integration. A number of methods have been developed to combine diverse datasets by removing technical batch effects, but most are computationally intensive. To overcome the challenge of enormous datasets, we have developed BBKNN, an extremely fast graph-based data integration algorithm. We illustrate the power of BBKNN on large scale mouse atlasing data, and favourably benchmark its run time against a number of competing methods.

**Availability and implementation:**

BBKNN is available at https://github.com/Teichlab/bbknn, along with documentation and multiple example notebooks, and can be installed from pip.

**Supplementary information:**

Supplementary data are available at *Bioinformatics* online.

## 1 Introduction

The past few years have seen a rapid development of single cell RNA-Seq, with its increased throughput allowing large scale atlas projects to release data for hundreds of thousands of cells (Tabula Muris Consortium *et al.*, 2018; Han *et al.*, 2018). As with any technology, variation in experimental procedures and conditions between labs creates batch effects that need to be corrected, especially if the potential of collaborative large scale atlasing efforts is to be realized (Kiselev *et al.*, 2018). A number of algorithms have been proposed to tackle this problem (Barkas *et al.*, 2018; Butler *et al.*, 2018; Haghverdi *et al.*, 2018; Hie *et al.*, 2019; Korsunsky *et al.*, 2018; Stuart *et al.*, 2019), but most of them struggle with excessive run time or resource requirements. This is likely to be further exacerbated as the size of scRNA-Seq collections continues to grow. The need for effective scaling into huge datasets is leading to scRNA-Seq analysis becoming established in Python, with SCANPY (Wolf *et al.*, 2018) offering a comprehensive set of analysis and visualization tools covering the entirety of a typical workflow. The only batch correction method currently operating in Python is Scanorama (Hie *et al.*, 2019), which has massive resource requirements that make it challenging to analyze large data collections.

Here, we present BBKNN (batch balanced k nearest neighbours), a simple, fast and lightweight batch alignment method. Performing batch correction at the neighbourhood graph inference step allows for the creation of an algorithm one to two orders of magnitude faster than existing methods, including those implemented with efficient performance in mind. BBKNN is written in Python and compatible with SCANPY, and its output can be immediately used for dimensionality reduction (McInnes and Healy, 2018), clustering (Traag *et al.*, 2019) and pseudotime inference (Haghverdi *et al.*, 2016). We illustrate the method’s utility using a large collection of mouse atlasing data (Tabula Muris Consortium *et al.*, 2018; Dahlin *et al.*, 2018; Deng *et al.*, 2014; Han *et al.*, 2018; Kernfeld *et al.*, 2018; Mohammed *et al.*, 2017; Park *et al.*, 2018; Zeisel *et al.*, 2015), and benchmark its run time against established methods on datasets of up to 2^19^ cells.

## 2 Materials and methods

A common step in scRNA-Seq analysis is the identification of a neighbourhood graph, often done as identifying each cell’s *k* nearest neighbours in principal component space. This graph is a good approximation of cell population structure, providing a basis for diverse downstream analysis. This includes clustering (Traag *et al.*, 2019), dimensionality reduced visualization (McInnes and Healy, 2018) and pseudotime trajectory inference (Haghverdi *et al.*, 2016). However, experimental variation added by batch effects often leads to cells being unable to connect to the same cell type/state across batches, introducing distortion and fracturing to this graph structure. This causes significant problems in all downstream analysis options outlined above.

BBKNN modifies the neighbourhood construction step to produce a graph that is balanced across all batches of the data. This approach treats the neighbour network as the primary representation of the data. For each cell, the BBKNN graph is constructed by finding the *k* nearest neighbours for each cell in each user-defined batch independently, resulting in each cell having an independent pool of neighbours in each batch. The neighbour sets are subsequently merged and processed via the UMAP algorithm (McInnes and Healy, 2018), which is the standard adopted by SCANPY (Wolf *et al.*, 2018). BBKNN’s speed stems from a combination of the simplicity of the algorithm with the default use of approximate neighbour detection (annoy, https://github.com/spotify/annoy). This allows the algorithm’s run time to linearly scale with cell total increase. An exact neighbour detection algorithm (Johnson *et al.*, 2017) is supported at a performance loss.

BBKNN’s main assumption is that at least some cells of the same type exist across batches, and that the differences between the same cell type across batches caused by batch effects are less than the differences between cells of different types within a batch. This is the core assumption of mnnCorrect (Haghverdi *et al.*, 2018) and other methods inspired by it. In this case, the graph construction will group together similar cell types across batches while leaving unrelated cell types well separated. Further details of the method, along with a demonstration on simulated (Zappia *et al.*, 2017) and real (Kiselev *et al.*, 2018) data, are discussed in the Supplementary Methods and Supplementary Figures S1–S4.

## 3 Results

Recent times have seen a veritable flood of murine scRNA-Seq data, with multiple labs across the world collecting diverse datasets ranging from early embryo development to fully matured adult organs. We have collated eight of those, covering cells from at least 26 different mouse organs (Tabula Muris Consortium *et al.*, 2018; Dahlin *et al.*, 2018; Deng *et al.*, 2014; Han *et al.*, 2018; Kernfeld *et al.*, 2018; Mohammed *et al.*, 2017; Park *et al.*, 2018; Zeisel *et al.*, 2015). After down-sampling the data to ensure balanced population sizes (Supplementary Methods, Supplementary Fig. S5), we ended up with a collection of 114 600 cells that were clearly split based on dataset of origin (Supplementary Fig. S6A). Applying BBKNN to the data overcomes this technical effect. Annotating the cells based on atlas of origin along with canonical marker genes (Supplementary Fig. S7) reveals an intuitive biological trajectory (Supplementary Fig. S6B). It starts in the centre of the manifold with embryonic stem cells, which branch into T cell, B cell, myeloid, megakaryocyte and erythrocyte populations in the top of the manifold and epithelial, mesenchymal, endothelial, muscular and neuronal cells in the other path. As such, not only does BBKNN successfully correct the batch effect, it manages to propose a biologically sound structure to the neighbour graph that translates to a cohesive trajectory in UMAP space. When correcting the same data with Harmony (Korsunsky *et al.*, 2018), the leading method in the field, cell populations are successfully merged but the final manifold is more fragmented, with no way to reconstruct the developmental trajectory (Supplementary Fig. S8). The quality of batch mixing in the corrected manifolds was assessed with kBET (Büttner *et al.*, 2019), with BBKNN mildly outperforming Harmony on average score (Supplementary Fig. S9).

In order to comprehensively evaluate BBKNN’s efficiency with relation to established methods (Barkas *et al.*, 2018; Butler *et al.*, 2018; Haghverdi *et al.*, 2018; Hie *et al.*, 2019; Korsunsky *et al.*, 2018; Stuart *et al.*, 2018), we used simulated data (Zappia *et al.*, 2017) to benchmark the algorithms on variably sized datasets (Supplementary Fig. S10). The total cell count was scaled in powers of two, from 2^11^ to 2^19^, with each dataset featuring two equally sized batches of two matching cell types. BBKNN’s default approximate neighbour mode scales linearly with the dataset increase and remains consistently one to two orders of magnitude faster than the other methods. The supported exact nearest neighbour algorithm does not scale linearly with dataset increase, but remains faster than Harmony across the benchmark. The other R-based approaches were left out at the 2^15^ mark, and Scanorama was unable to complete processing the 2^16^ cell dataset due to resource constraints. The benchmarking was carried out on a personal MacBook Pro with 16GB RAM and a four-core i7 processor.

## Funding

This project was supported by Wellcome Sanger core funding (no. WT206194) and Wellcome grant (211276/Z/18/Z). J.-E.P. is supported by an EMBO Long-Term Fellowship. Z.M. is supported by a Single Cell Gene Expression Atlas grant from the Wellcome Trust (nr. 108437/Z/15/Z).

*Conflict of Interest*: none declared.

## Supplementary Material

btz625_Supplementary_Data
