# Supplementary material for: BBKNN: fast batch alignment of single cell transcriptomes
Source: Bioinformatics. 2019 Aug 10;36(3):964–5. doi: 10.1093/bioinformatics/btz625 (PMC9883685; doi:10.1093/bioinformatics/btz625)
Supplement: btz625_Supplementary_Data [file bioinformatics_36_3_964_s1.pdf]

Supplementary Methods

Detailed Description of the BBKNN Method

BBKNN is designed to use the network representation of scRNA-Seq data as the primary representation of the data. To this end, the aim of the BBKNN method is to construct the network in such a way that cells of the same type or state are connected across batches (Supplementary Figure 1). Let the number of UMIs observed for a gene g in cell c be given by x_{gc}. Data preparation for BBKNN first normalises each cell for depth of sequencing and applies a variance stabilising transformation to produce a normalised measure of expression, y_{gc}, given by

$$y_{gc} = \log(1 + f \frac{x_{gc}}{\sum_g x_{gc}}) \quad (1)$$

where f is a scaling factor with a default value of 10000.

The next step of the method is to identify neighbours both within and between batches. In theory this can be performed directly on the normalised data y_{gc} using any valid distance metric. In practice, we follow what has emerged as the standard practice in the field [5, 16] and calculate the principal component transformation of $y_{gc} \forall c$ and $g \in \Omega$, where Ω is a set of genes chosen to have high variability. Finally, we consider only the first N principal components to represent the data, which we represent as z_{pc} for component p, cell c. Transforming and truncating the data in this way helps alleviate the curse of dimensionality [1, 4] and ignores variability that is likely due to random noise.

Given some normalised representation of the expression data for each cell c, such as z_{pc}, and a distance metric d, we then calculate the k nearest neighbours for each cell *within each batch*. That is, for each cell c and batch b we find the set of cells,

$$S_{bc} = \{a \in C_b | d(z_c, z_b) \leq \delta_{bc}\} \quad (2)$$

where C_b is the set of cells belonging to batch b, $d(z_c, z_b)$ gives the distance between cells b and c and δ_{bc} is the distance to the kth furthest cell in C_b from cell c. That is, S_{bc} is the set of k cells in batch b that are closest to cell c. The complete set of neighbours for a cell c, which we denote S_c, is given by

$$S_c = S_{b_0 c} \cup S_{b_1 c} \cup \cdots S_{b_n c} \quad (3)$$

We use angular distance as the default when computing approximate neighbours as it is the preferred metric of annoy [3], and Euclidean distance as the default when computing exact neighbours. Other metrics are supported, often at a performance loss. In addition to returning a list of neighbours, the KNN algorithms also provide pairwise distances between neighbours, which we then use in calculating the weights for network edges as described below.

Calculating $S_c \forall c \in C$ provides a connected graph representation of the data with each cell connected to its k nearest neighbours within and between batches. However, a number of these connections will be undesirable in the

sense that they connect unrelated cell types. To account for this, we use the approach taken by the UMAP method [11] and calculate a connectivity score for each pair of connected points. Specifically, for each cell c, we calculate,

$$\alpha_{ca} = exp\left(-\frac{(d(z_c, z_a) - \rho_c)}{\sigma_c}\right) \tag{4}$$

where $\rho_c = min(d(z_c, z_a), a \in S_c)$ and σ_c is chosen to satisfy,

$$\sum_{a \in S_c} \alpha_{ca} = \lambda \log_2(|S_c|) \tag{5}$$

where λ is a bandwidth parameter (set to 1 by default) that controls how quickly the connectivity values decay to 0 with distance.

The connectivity score α_{ca} is then made symmetric to give,

$$w_{ca} = w_{ac} = \alpha_{ac} + \alpha_{ca} - \alpha_{ac}\alpha_{ca} \tag{6}$$

Each connection between cells is given the corresponding weight, w_{ca}, producing a weighted network representation of the data.

k is a free parameter that must be specified with lower values emphasising local structure and larger values emphasising global structure. As a general rule of thumb, we set k such that $k * N_b \approx 30$ where N_b is the number of batches in the experiment. An exception to this heuristic rule is when N_b becomes large. In such circumstances it is desirable to prevent k falling below 3 so that the within batch neighbour structure is adequately sampled. To prevent the global neighbour number, $k * N_b$, becoming too large in these situations we globally trim the neighbour network by calculating

$$T_c = \{a \in S_c | w_{ca} \geq \epsilon_c\} \tag{7}$$

where ϵ_c is the tth largest connectivity score in the set S_c. That is, T_c is a reduced set of the t nearest neighbours of cell c, where nearest is defined using the connectivity scores. T_c is then used instead of S_c to construct the weighted graph representation of the data.

This trimming allows the local batch structure of each cell to be adequately sampled without compromising the locality of the neighbour network constructed from combining batch specific neighbours. Under certain well justified assumptions, this weighted network representation of the data strongly connects cells of the same type that have been separated by batch, while leaving unrelated cell types separate. Furthermore, under the same assumptions the trimming process outlined in equation 7 will preferentially remove connections between unrelated cell types connected across batches (see the following section).

Finally, while this weighted network can be used as input for many downstream analyses, certain methods require an *unweighted* network. In such situations the trimmed network defined by T_c can simply be used without weights.

Theoretical Justification for the BBKNN Algorithm

The core assumption made by BBKNN is that differences between cell types are greater than differences between batch. This is an assumption that is also made by other batch correction methods, most notably mnnCorrect [6]. To make this more precise, consider two cells α and β. There are four possible situations for these cells, specifically:

1. d_1 - α and β are the same cell type and from the same batch.

2. d_2 - α and β are the same cell type and from different batches.

3. d_3 - α and β are different cell types and from the same batch.

4. d_4 - α and β are different cell types and from different batches.

the assumption that batch-to-batch variation is less than cell type to cell type variation implies that on average, $d_1 < d_2 < d_3 < d_4$. As such, the nearest neighbour of a cell within another batch will always be of the same cell type (as $d_2 < d_4$), provided the cell type is present in both batches.

Therefore, if the same cell type is present in multiple batches, BBKNN will always create a connection between these cells. However, there will be situations in which a cell type is present in only a subset of batches. In this case, the requirement to include neighbours from all batches will force BBKNN to connect different cell types across batches. For example, consider a cell type that exists in only one batch. BBKNN will then connect a cell of this type to whatever cell of another type happens to be closest in other batches in the experiment.

However, such connections are automatically de-emphasised by the weighting step of BBKNN for two reasons. Firstly, the cell connectivity scores α_{ac} decay exponential with distance, with the scale of this decay set by equation 5. Equation 5 is calculated using all neighbours for cell c across all batches. As such, it will be calculated using distances to cells of the same type in the same batch (case 1 above) as well as the erroneous cross batch, cross cell type distances (case 4). The assumption that $d_1 < d_4$ implies that σ_c must be small and the connectivity values to cells of different types in different batches must be small as well.

The second reason cross cell type, cross batch connections are automatically de-emphasised by BBKNN is due to the symmetrised connectivity scores w_{ac} given by equation 6. As cells connected across type and batch are unlikely to be mutual neighbours [6], the symmetrised values w_{ac} used as weights will be little greater than their asymmetric components. That is, one of α_{ac} and α_{ca} is likely to be zero when c and a are not of the same cell type.

The global trimming of the neighbour network described in equation 7 provides a final level of protection against connections between unrelated cells across batches. As this procedure preferentially removes neighbours based on their connectivity scores, it will tend to remove connections from case 4 to case 1 above. That is, $d_4 > d_2 \Rightarrow w_4 < w_2$ and so the cells with the lowest connectivity scores, that are removed first by the trimming procedure, will be those that

connect cells of different type across (and within) batches. By default, BBKNN trims the graph to allow a number of top connectivities equal to ten times the total number of neighbours computed for each cell, but this can be controlled by an input parameter. More stringent trimming helps increase population independence at the potential cost of quality of batch mixing, emphasising local structure over the global one.

In short, BBKNN creates connections between cells of different types when batch specific cell types are present. However, these connections are not a problem for the method for the same reason that connections between different cell types are not a problem for the UMAP method. Specifically, as the distance between cell types is greater than the distance within cell types, the calculation of weights for the neighbour graph automatically de-emphasises these connections.

BBKNN Implementation

BBKNN is implemented in Python, and is designed to slot into the spot occupied by neighbourhood graph inference in the typical SCANPY workflow, making use of PCA coordinates stored within the AnnData object. For non-SCANPY users, a second function taking a PCA matrix and batch assignment vector on input is provided. The neighbour identification itself is performed via annoy [3]. Exact neighbours can be identified at a performance loss via faiss [7] for the Euclidean metric. cKDTree [10] from the spatial module of scipy [8] is provided as a slower backup due to faiss's demanding setup requirements. Additional exact neighbour metrics are supported by KDTree from the neighbors module of sklearn [13] at further reduced performance.

Seurat-Inspired SCANPY Workflow

All analysis scenarios were evaluated using a common analysis core, which shall be henceforth referred to as the Seurat-inspired SCANPY workflow. The steps of the analysis are normalising the data to 10,000 counts per cell, identifying highly variable genes, limiting the datasets to those genes only, log transforming the data, scaling it to unit variance and zero mean followed by PCA. At this stage, the established analysis identifies a regular KNN graph, but we also apply BBKNN in parallel. Both resulting AnnData objects are subsequently dimensionality-reduced with UMAP and are subjected to graph-based clustering.

Ground Truth Batch Simulation

BBKNN's ability to merge cell types from matching populations, along with the impact of trimming on the distinctness of unrelated cell groups, was assessed using Splatter [17] simulated data. The dataset was created with three 500 cell batches, each formed of three equally common cell types. 5000 genes

were simulated, and both batch effect control parameters were increased to 0.5 to increase the severity of the effect. The data was processed using the Seurat-inspired SCANPY workflow. Supplementary Figure 2 demonstrates the heavy batch effect present when processing the data with the standard KNN procedure, with BBKNN successfully recovering the buried cell types.

The impact of graph trimming on unrelated cell populations is examined in Supplementary Figure 3, with two of the original simulation batches kept and one of them having a cell type removed. This creates a setup with two populations present in both batches, and one cell type being specific to a single batch. Initially, BBKNN places the isolated population near an unrelated cell type, and this effect persists with the default trimming value. Trimming the graph to a narrower set of top connectivities for each cell restores the autonomy of the population, illustrating the need to inspect the proposed manifold for any erroneously merged populations and adjust the trimming parameter accordingly.

Pancreatic Data

The impact of the trimming parameter was further inspected on real data in Supplementary Figure 4, using four pancreatic datasets [2, 12, 14, 15] collated and uniformly annotated as part of the scmap resource [9]. The data was processed with the Seurat-inspired SCANPY workflow, revealing a major batch effect in the uncorrected manifold which gets rectified by BBKNN. However, when no trimming is performed, the endothelial population becomes erroneously connected to the main cell manifold. This effect is milder than in the simulated case, and is removed by the default level of trimming. When performing more stringent trimming matching that necessary to isolate the unrelated populations in the simulated data, the cell types remain separated to a similar standard. However, the quality of the batch integration suffers due to the prioritising of local structure.

Murine Atlases

Both the droplet and plate data from Tabula Muris was downloaded from figshare, while all the other atlases were obtained from GEO. The dataset was then analysed with the Seurat-inspired SCANPY workflow (Supplementary Figure 5). To avoid cell type over-representation biases, in particular due to a disproportionately large hematopoietic stem cell population, the dataset was downsampled based on organ annotations provided within each atlas coupled with Louvain clustering of the complete object with a regular KNN graph – if a given atlas featured over 2,000 cells of a given organ, the cluster memberships of all of those cells were extracted. If any cluster featured over 500 cells from that organ in that atlas, those cells would be randomly sub-sampled to remove the excess. The Seurat-inspired SCANPY workflow was repeated after downsampling (Supplementary Figure 6). Cell populations were annotated based on canonical markers (Supplementary Figure 7). Harmony was ran on the

same PCA space as BBKNN (Supplementary Figure 8). The quality of BBKNN and Harmony correction in UMAP space was assessed with kBET on a per-population level, with lower scores implying a better degree of batch correction (Supplementary Figure 9). The methods perform to a similar standard, with BBKNN having a minor advantage in average score (0.9468 to 0.9646, lower being better). A notable population is muscle, with the kBET scores differing by 0.2 in BBKNN's favour.

Simulated Data Benchmarking

The run time of BBKNN was compared to mnnCorrect, Seurat 2 and 3's CCA-based batch correction approaches, Conos, Scanorama and Harmony using Splatter simulated data (Supplementary Figure 10). Both approximate and exact BBKNN neighbour detection algorithms were included in the benchmark. The total dataset size increased in powers of two from 2^{11} ($\sim 2,000$) to 2^{19} ($\sim 500,000$) cells, with the formal simulation design being 5000 gene datasets of two equally sized batches, each with two cell types. mnnCorrect, the Seurat methods, Conos and Scanorama were ran on the resulting count matrix, while BBKNN and Harmony used a PCA representation on input. Scanorama ran into resource constraints when processing the 2^{16} ($\sim 65,000$) cell dataset. From that point onward, only BBKNN and Harmony were ran, and the simulated gene pool was shrank to 500 on account of both methods operating on PCA coordinates. As such, the simulated gene total has no impact on the run time of these algorithms. The benchmarking was performed on a four-core i7 MacBook Pro with 16GB RAM.

References

[1] Charu C. Aggarwal, Alexander Hinneburg, and Daniel A. Keim. On the surprising behavior of distance metrics in high dimensional space. In Jan Van den Bussche and Victor Vianu, editors, *Database Theory — ICDT 2001*, pages 420–434, Berlin, Heidelberg, 2001. Springer Berlin Heidelberg.

[2] Maayan Baron, Adrian Veres, Samuel L Wolock, Aubrey L Faust, Renaud Gaujoux, Amedeo Vetere, Jennifer Hyoje Ryu, Bridget K Wagner, Shai S Shen-Orr, Allon M Klein, et al. A single-cell transcriptomic map of the human and mouse pancreas reveals inter-and intra-cell population structure. *Cell systems*, 3(4):346–360, 2016.

[3] Erik Bernhardsson. Annoy: Approximate nearest neighbors in c++/python optimized for memory usage and loading/saving to disk, 2013. *URL https://github. com/spotify/annoy*, 2013.

[4] Kevin S. Beyer, Jonathan Goldstein, Raghu Ramakrishnan, and Uri Shaft. When is "nearest neighbor" meaningful? In *Proceedings of the 7th In-*

ternational Conference on Database Theory, ICDT '99, pages 217–235, London, UK, UK, 1999. Springer-Verlag.

[5] Andrew Butler, Paul Hoffman, Peter Smibert, Efthymia Papalexi, and Rahul Satija. Integrating single-cell transcriptomic data across different conditions, technologies, and species. *Nature biotechnology*, 36(5):411, 2018.

[6] Laleh Haghverdi, Aaron TL Lun, Michael D Morgan, and John C Marioni. Batch effects in single-cell rna-sequencing data are corrected by matching mutual nearest neighbors. *Nature biotechnology*, 36(5):421, 2018.

[7] Jeff Johnson, Matthijs Douze, and Hervé Jégou. Billion-scale similarity search with gpus. *arXiv preprint arXiv:1702.08734*, 2017.

[8] Eric Jones, Travis Oliphant, Pearu Peterson, et al. Scipy: open source scientific tools for python. 2001.

[9] Vladimir Yu Kiselev, Andrew Yiu, and Martin Hemberg. scmap: projection of single-cell rna-seq data across data sets. *Nature methods*, 15(5):359, 2018.

[10] Songrit Maneewongvatana and David M Mount. Analysis of approximate nearest neighbor searching with clustered point sets. *Data Structures, Near Neighbor Searches, and Methodology*, 59:105–123, 2002.

[11] Leland McInnes and John Healy. Umap: Uniform manifold approximation and projection for dimension reduction. *arXiv preprint arXiv:1802.03426*, 2018.

[12] Mauro J Muraro, Gitanjali Dharmadhikari, Dominic Grün, Nathalie Groen, Tim Dielen, Erik Jansen, Leon van Gurp, Marten A Engelse, Francoise Carlotti, Eelco JP de Koning, et al. A single-cell transcriptome atlas of the human pancreas. *Cell systems*, 3(4):385–394, 2016.

[13] Fabian Pedregosa, Gaël Varoquaux, Alexandre Gramfort, Vincent Michel, Bertrand Thirion, Olivier Grisel, Mathieu Blondel, Peter Prettenhofer, Ron Weiss, Vincent Dubourg, et al. Scikit-learn: Machine learning in python. *Journal of machine learning research*, 12(Oct):2825–2830, 2011.

[14] Åsa Segerstolpe, Athanasia Palasantza, Pernilla Eliasson, Eva-Marie Andersson, Anne-Christine Andréasson, Xiaoyan Sun, Simone Picelli, Alan Sabirsh, Maryam Clausen, Magnus K Bjursell, et al. Single-cell transcriptome profiling of human pancreatic islets in health and type 2 diabetes. *Cell metabolism*, 24(4):593–607, 2016.

[15] Yue J Wang, Jonathan Schug, Kyoung-Jae Won, Chengyang Liu, Ali Naji, Dana Avrahami, Maria L Golson, and Klaus H Kaestner. Single cell transcriptomics of the human endocrine pancreas. *Diabetes*, page db160405, 2016.

[16] F Alexander Wolf, Philipp Angerer, and Fabian J Theis. Scanpy: large-scale single-cell gene expression data analysis. *Genome biology*, 19(1):15, 2018.

[17] Luke Zappia, Belinda Phipson, and Alicia Oshlack. Splatter: simulation of single-cell rna sequencing data. *Genome biology*, 18(1):174, 2017.

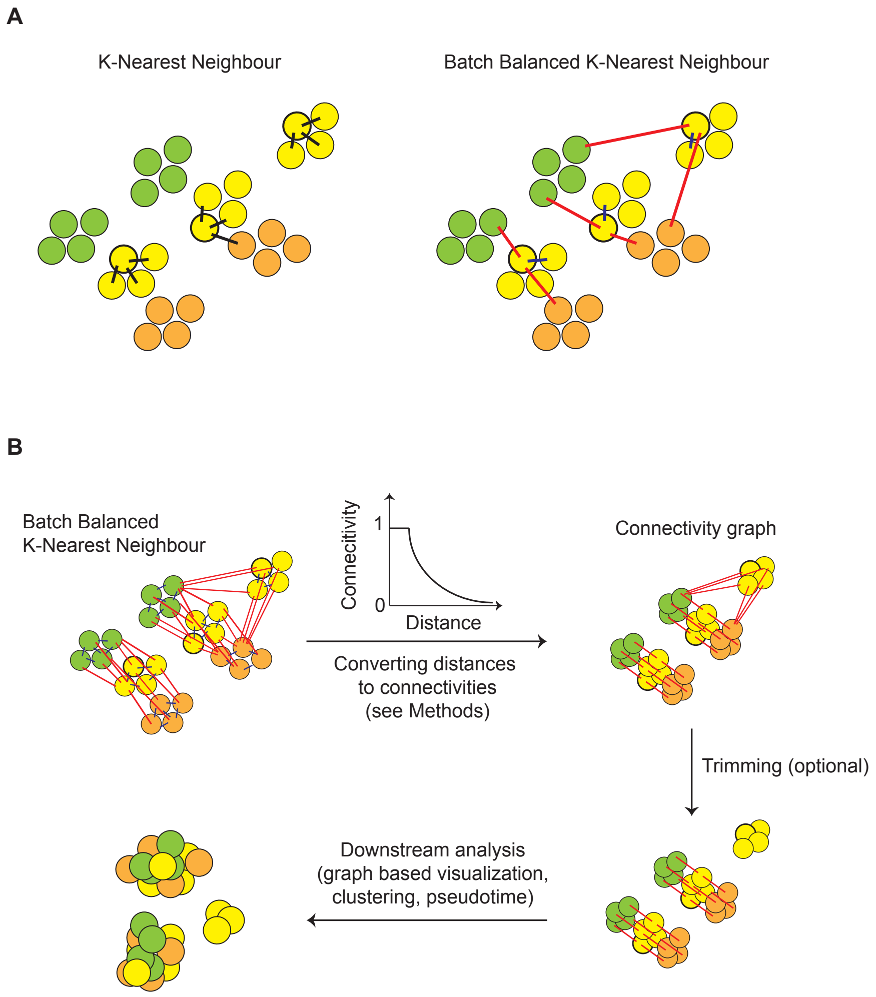

Supplementary Figure 1: A conceptual schematic of BBKNN's operation. Identifying a cell's k nearest neighbours for the purpose of constructing a KNN graph compared to the batch balanced counterpart in BBKNN (A). The neighbour distance collection is then converted to exponentially related connectivities, which BBKNN trims to weed out any erroneous connections between unrelated cell populations (B). This connectivity graph can serve as the basis for a number of downstream analysis options.

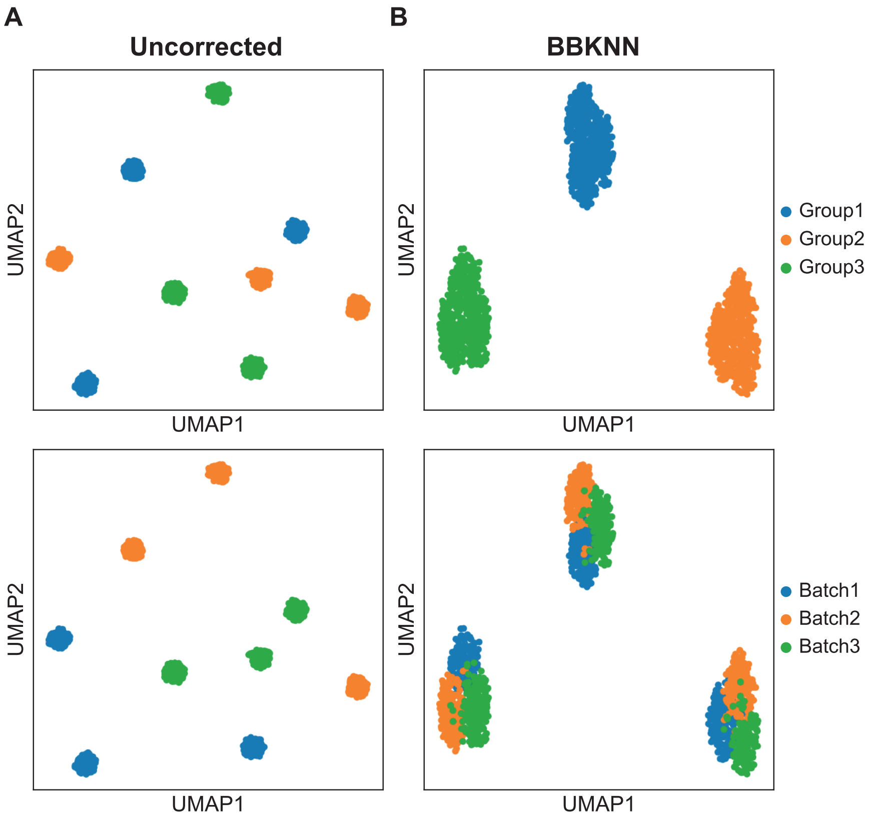

Supplementary Figure 2: Assessing BBKNN's functionality on simulated data. Three batches of three cell types were generated using Splatter, and analysing them without batch correction leads to the visualisation being distorted by the technical effect (A). Applying BBKNN manages to correctly reconnect the cell types across the batches (B).

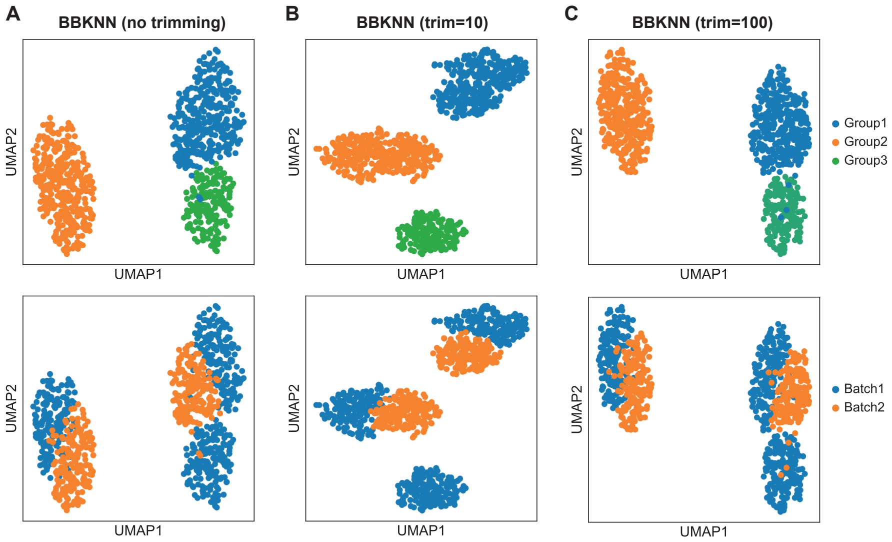

Supplementary Figure 3: Applying BBKNN trimming to simulated data. A simulation scenario purposefully confuses BBKNN by including a cell type in only one of the batches, with the algorithm placing it near an unrelated population (A). Trimming the neighbourhood graph to a narrow set of top connectivities for each cell correctly recovers this cell type (B). BBKNN's default trimming threshold is too lenient to separate the populations as the effect is severe (C).

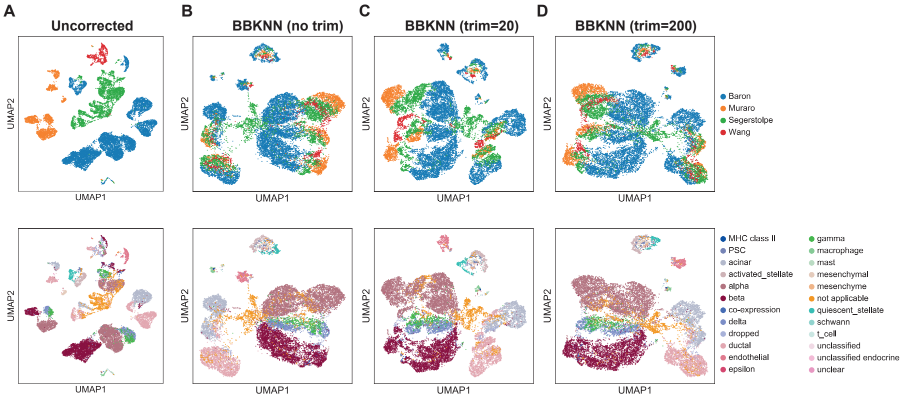

Supplementary Figure 4: A collection of four experimentally diverse pancreatic datasets with a shared biology, confounded by a severe batch effect (A). BBKNN is able to successfully reconnect matching cell types, but the endothelial population is connected to the main manifold when no trimming is performed (B). Performing trimming matching the stringent (C) and lenient (D) thresholds from the simulated analysis successfully disconnects the population, with the stringent threshold's emphasis on local structure leading to more conserved batch effect.

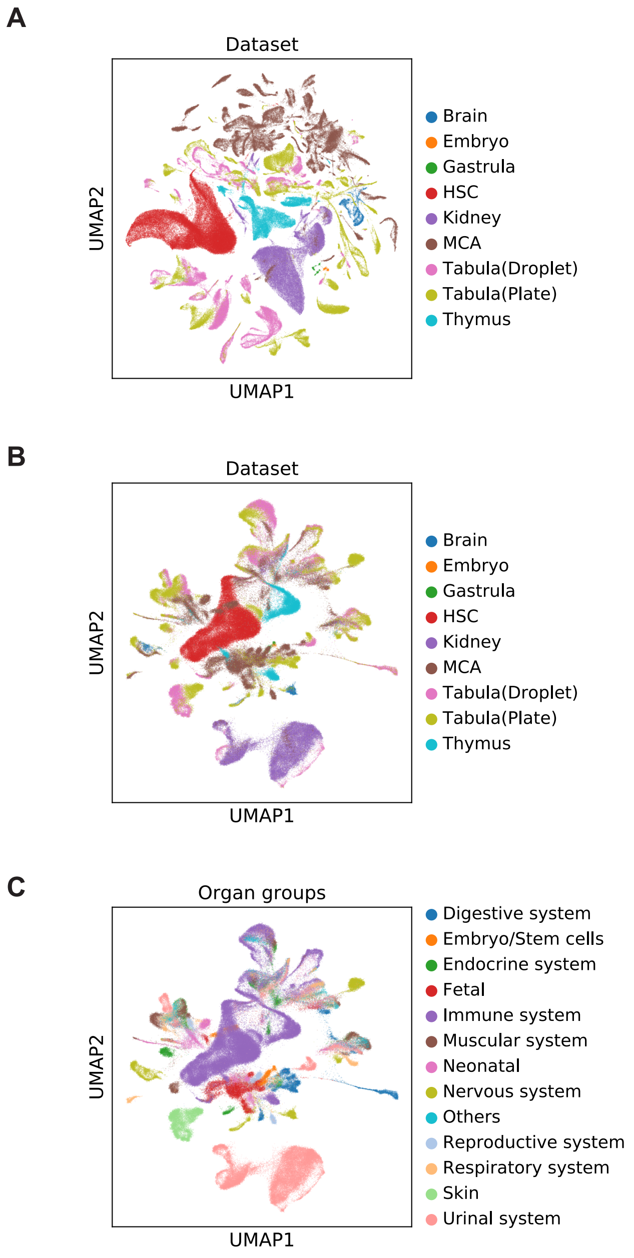

Supplementary Figure 5: Analysing the complete 267,690 cell murine atlas collection. Merging all of the data sources leads to a clear divide based on the study of origin (A), which is successfully amended by BBKNN (B,C).

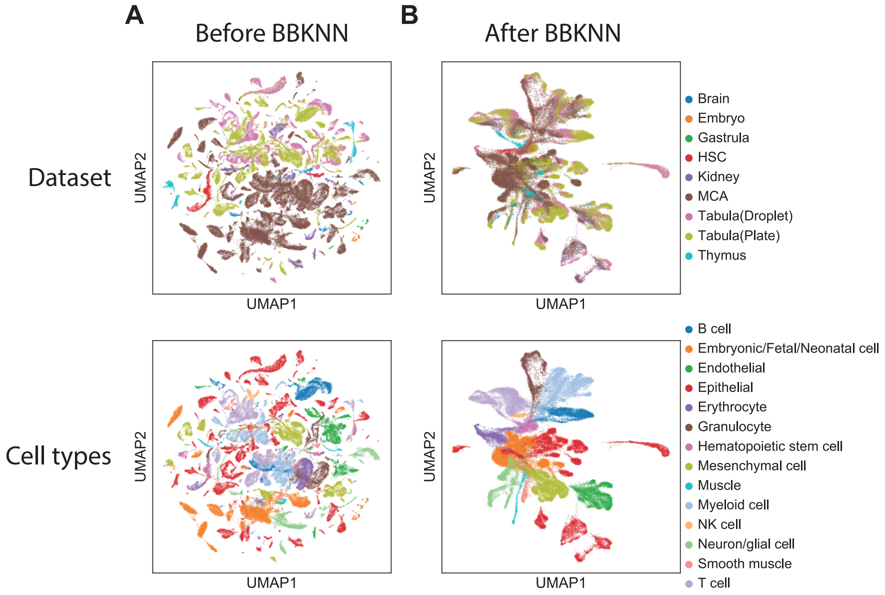

Supplementary Figure 6: Repeating the murine atlas collection analysis after down-sampling to ensure more balanced population sizes. The clear technical effect (A) is removed by BBKNN (B), and the final manifold captures a biological trajectory from embryonic cells to fully mature immune/non-immune populations.

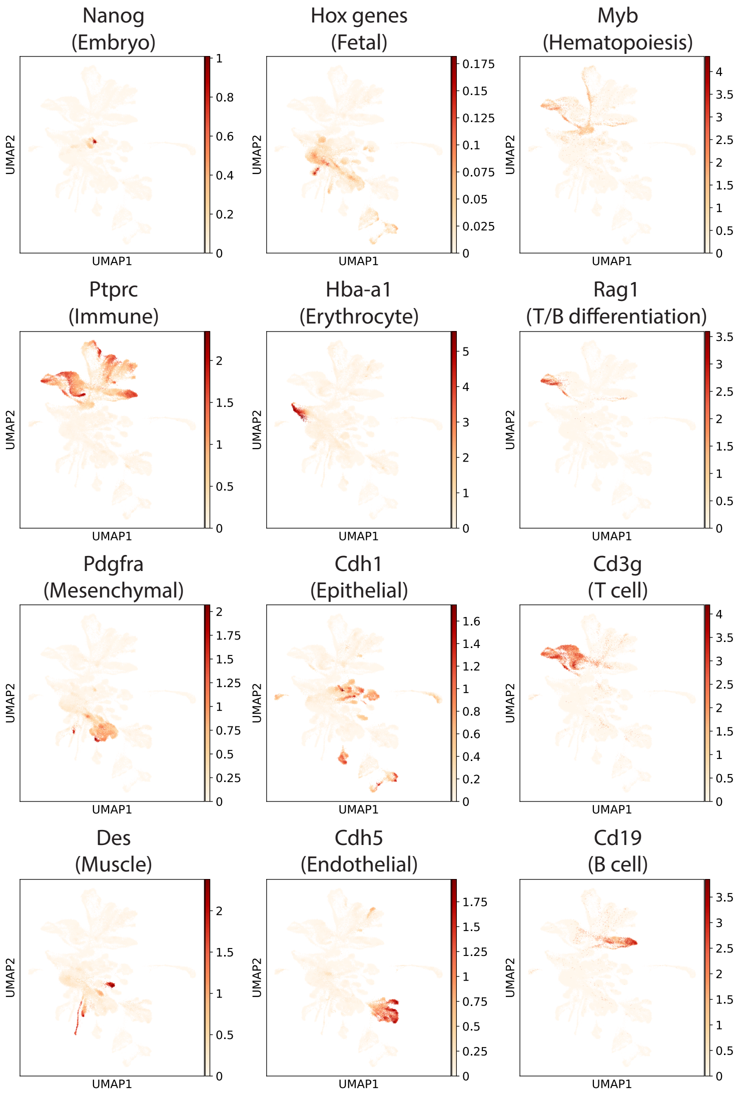

Supplementary Figure 7: The expression of a collection of known canonical markers in the merged murine atlas manifold. These genes were used to annotate the featured cell populations.

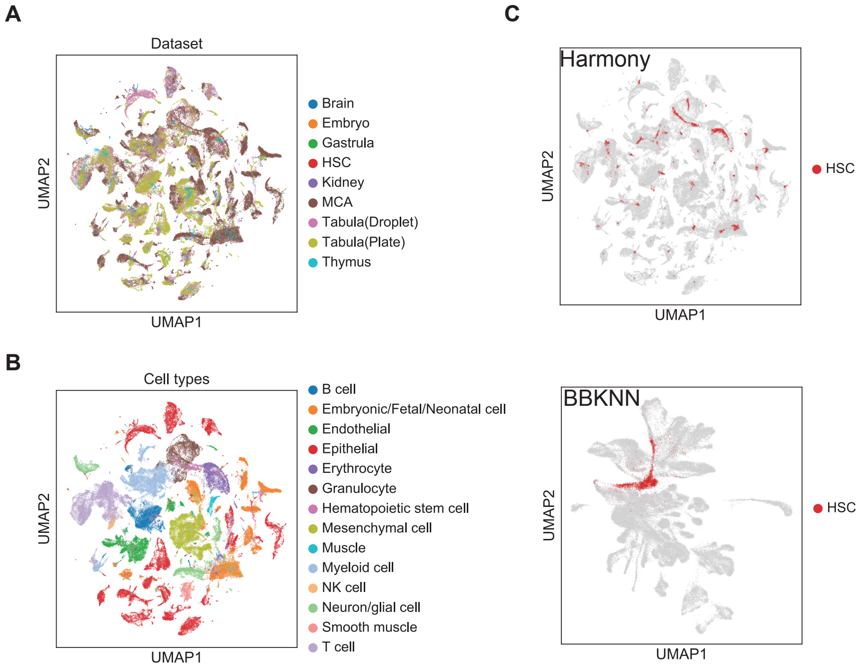

Supplementary Figure 8: Harmony batch correction of the murine atlases. The datasets are well mixed (A), and the cell types are successfully reconnected (B) in most cases. However, the resulting manifold is considerably more fragmented than the one proposed by BBKNN, with the purified hematopoetic stem cell population from the HSC dataset split across the whole space instead of forming a centralised hub (C).

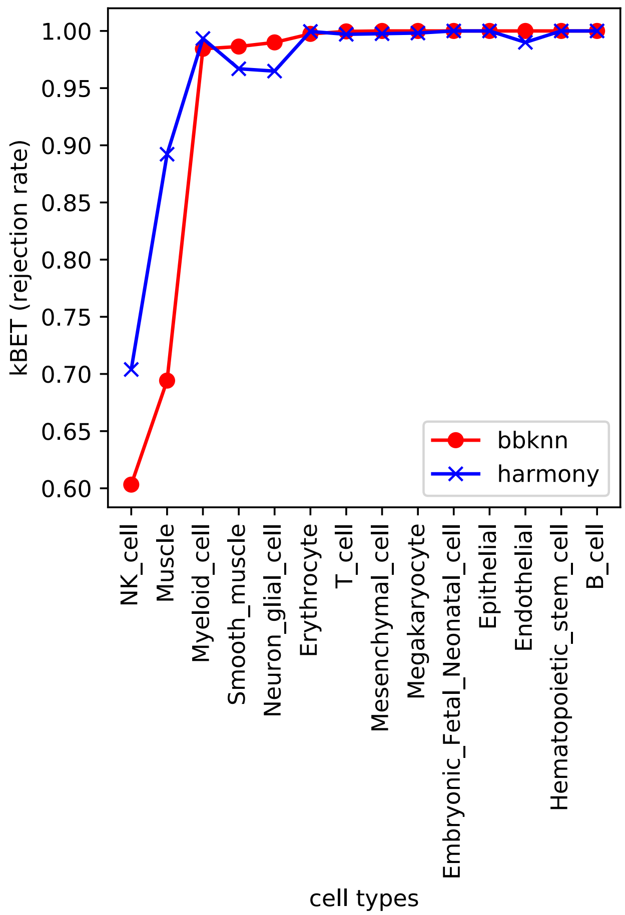

Supplementary Figure 9: Assessing the quality of BBKNN and Harmony's correction of the murine atlases by running kBET on UMAP space. The scores are computed on a per-population level, with lower values implying better batch correction. On average, BBKNN slightly outperforms Harmony.

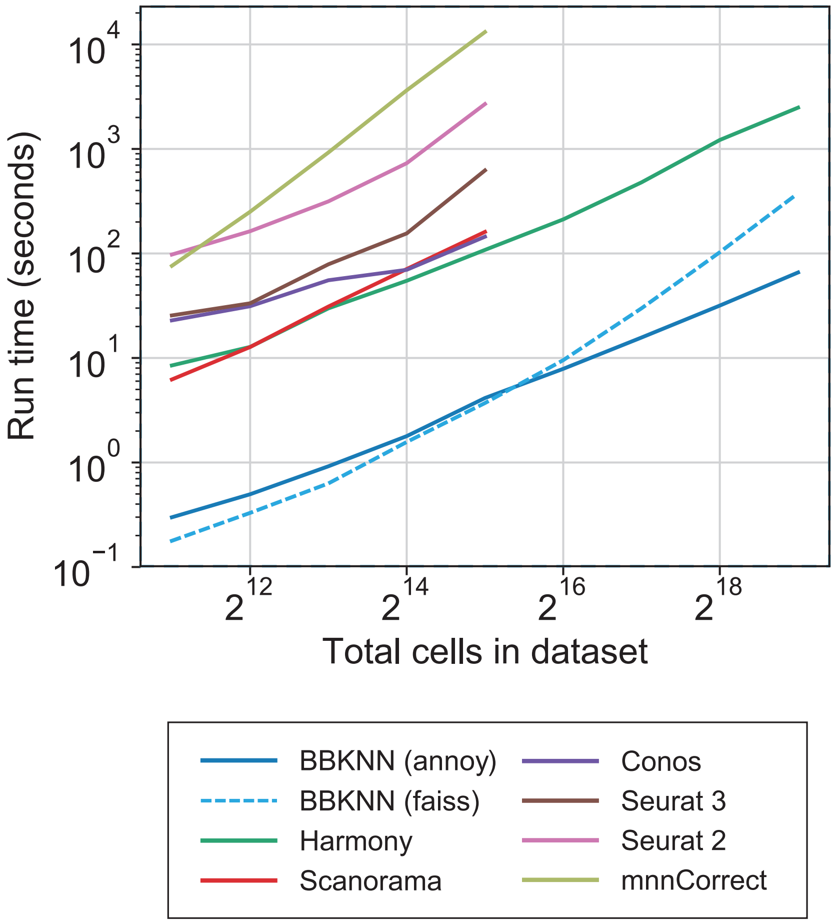

Supplementary Figure 10: Benchmarking the run time of BBKNN and a number of established batch correction methods using simulated data. BBKNN's run time compares favourably to that of established methods, with the algorithm being consistently one to two orders of magnitude faster over a wide range of input dataset sizes.
